# Fungal hydrophobins render stones impermeable for water but keep them permeable for vapor

**DOI:** 10.1038/s41598-019-42705-w

**Published:** 2019-04-18

**Authors:** Lex Winandy, Olexandra Schlebusch, Reinhard Fischer

**Affiliations:** Karlsruhe Institute of Technology (KIT) - South Campus, Department of Microbiology, Fritz-Haber-Weg 4, D-76131 Karlsruhe, Germany

**Keywords:** Fungi, Biomaterials

## Abstract

The conservation of architectural heritage is a big challenge in times with increasing air pollution with aggressive gases. A second major threat to buildings is the combination of water and air contaminants which may be used by microorganisms for their metabolism. Hence, myriads of different bacteria and fungi populate stone surfaces and penetrate into the fine pores and cracks. Whereas epoxid-based paintings (or other paintings) may protect the coated surfaces from water and aggressive gases, these chemicals seal the stone surface and prevent also the evaporation of vapor from the inside of the buildings. Here, we tested a natural, fungal protein-based coating method. Fungi use small, amphiphilic proteins to turn their surfaces hydrophobic. We found that *Aspergillus nidulans* hydrophobin DewA and *Trichoderma reesei* HFBI confer hydrophobicity to stones but keep their pores open. The effect resembles “Gore-tex” fabric material.

## Introduction

One of the most important challenges in the conservation of architectural elements made of stone is the protection against water^1^. Multiple freeze/thawing cycles of water inside the stone bulk are mainly responsible for stone decay. Over the last decades, several treatments have been proposed to reduce water absorption by increasing the hydrophobicity of stone surfaces. One way to achieve water repellency is by increasing the roughness of the substrate surface^2,3^. The use of a plasma treatment, despite being successful^4^, has been rejected due to the special equipment and complex control processes involved^5^. Other methods are the application of different organic compounds like synthetic and natural waxes^6^, acrylic and siloxane resins^7,8^ and perfluoropolyethers^9^ which form water repellent films on the surface. In the last years, the addition of different nanoparticles to protective products was heavily investigated. Inorganic silica (SiO_2_) and titanium dioxide (TiO_2_) nanoparticles were added to PMMA (polymethylmethacrylate), polyalkylsiloxane^10,11^ or PDMS (polydimethylsiloxane)^12^ to further increase the polymer hydrophobicity or to confer self-cleaning properties to the coatings.

A class of organic “nanoparticles” heavily investigated in the past 15 years are hydrophobins. They are small amphiphilic proteins secreted by fungi to reduce water surface tension or to increase the hydrophobicity of aerial hyphae or conidiospores^13–15^. Hydrophobins can self-assemble into stable monolayers on hydrophilic and hydrophobic surfaces and change their wettability properties^13,16,17^. They can be divided into two classes [I and II] differing in size and monolayer stability. While class I hydrophobins are resistant to detergents and high temperatures, class II hydrophobins can be removed from surfaces with ethanol, detergents or pressure^18–20^. So far hydrophobins have been applied in surface coating and modification, foam and emulsion stabilization and the increase of enzyme activity^21–25^. The application involving water at neutral pH as non-toxic solvent and the possibility of complete removal from the surface make hydrophobins an interesting candidate for stone protection with respect to the Restauration Charters^26,27^ and requirements to protectives imposed by several authors^28,29^.

In this study we applied the class I hydrophobin DewA from *Aspergillus nidulans* and the class II hydrophobin HFBI from *Trichoderma reesei* on three different lithotypes. Obernkirchen sandstone, Balegem limestone and Carrara marble were coated with either hydrophobin and we characterized surface binding properties, penetration depth and water repellency. Additionally, vapor permeability and coating stability of the treatments were tested.

## Results

### Coating of different lithotypes

**Figure 1:**
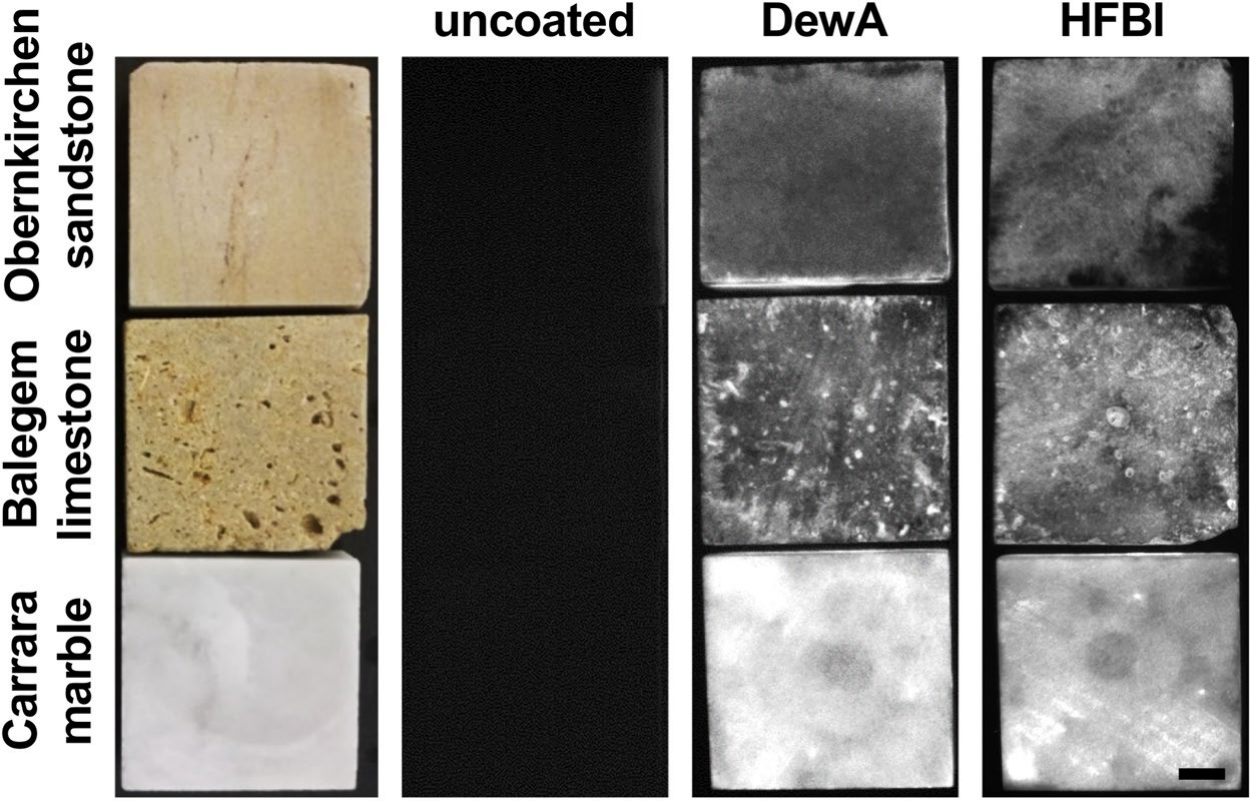
Coating of Obernkirchen sandstone, Balegem limestone and Carrara marble with hydrophobins DewA and HFBI. The left row shows specimens of the three lithotypes (5 × 5 × 1 cm blocks) and imaged in bright field. The three following rows show uncoated stone samples and with DewA and HFBI coated specimen after immune detection of hydrophobin. The successful coating of stone with hydrophobin is proven by light emission whereas the uncoated samples did not emit any light and appear dark. Scale bar = 1 cm.

**Figure 2:**
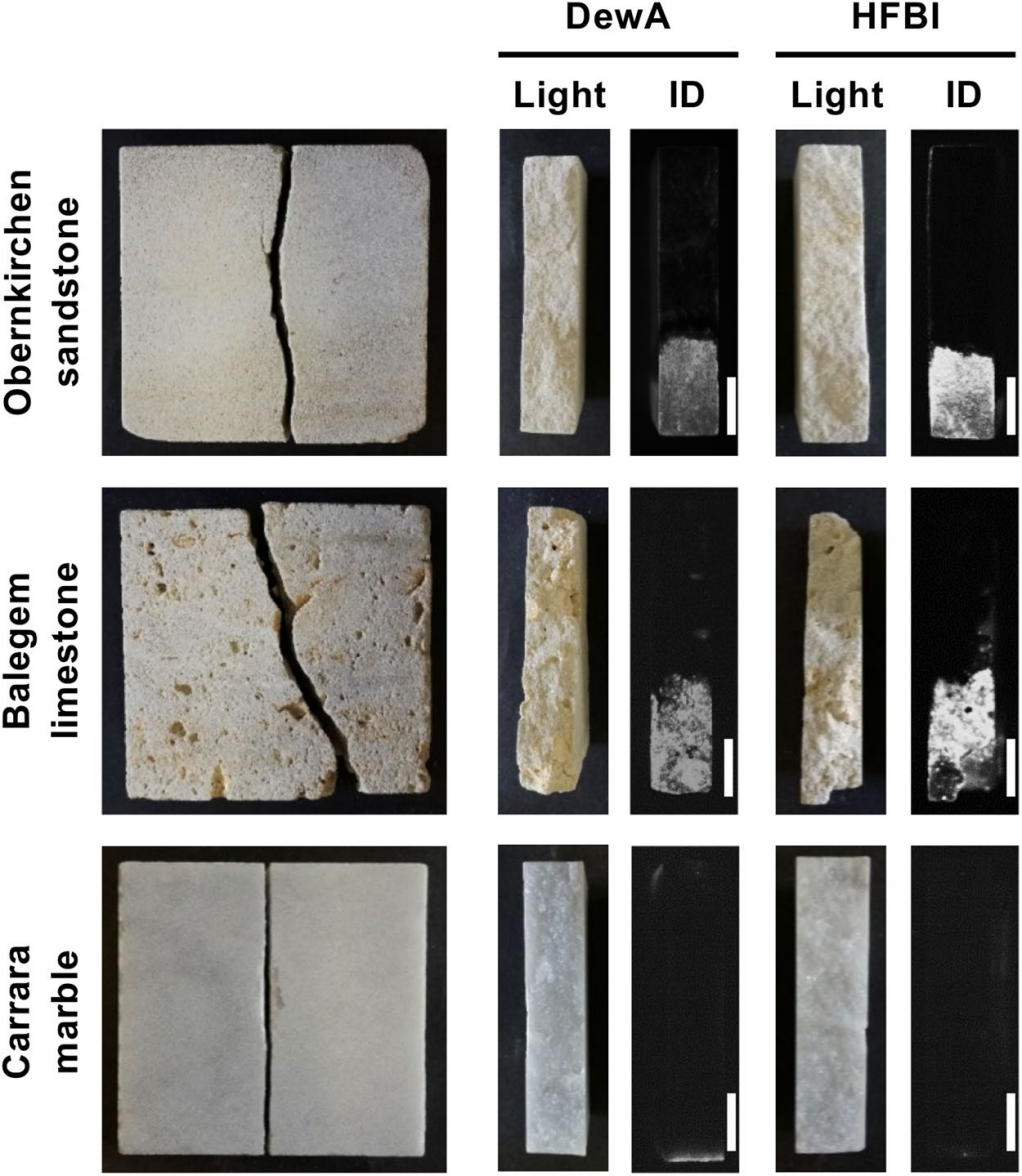
Penetration depth of hydrophobin coating. Stone specimens were placed with the narrow side into the hydrophobin solution and incubated for 2 h. After several washing steps and complete drying, the stone plates were cracked and hydrophobins visualized by immunodetection. Shown are the cracked stones in light and the immunodetection images (ID). Scale bar = 1 cm.

Nanoparticles are increasingly exploited in many research fields, one which is the conservation of deteriorated stones. Hydrophobin DewA from *A. nidulans* and HFBI from *T. reesei*, allocable to nanoparticles due to their small size, were produced in *E. coli* as described previously^30^. Samples of Obernkirchen sandstone, Balegem limestone and Carrara white marble were treated with solutions containing 100 µg/ml of the 6xHis-tagged hydrophobins DewA or HFBI. It has been shown before, that 100 µg/ml is a good concentration to obtain homogenous coatings. Higher hydrophobin concentrations lead to aggregations of the protein^30,31^. Positive hydrophobin coating was visualized by immunodetection and compared to untreated stone samples. Both hydrophobins were able to coat the three different lithotypes (Fig. 1). The coating appeared homogenous. To test how deep hydrophobins can penetrate the stone, 5 cm × 5 cm × 1 cm stone specimens were placed vertically into a hydrophobin solution for two hours. After the incubation and complete drying, the specimens were split and hydrophobin penetration depth was visualized by immunodetection. DewA and HFBI penetrated ca. 1 cm in Obernkirchen sandstone and up to 1.5 cm in Balegem limestone. Hydrophobins were unable to permeate significantly into marble (Fig. 2).

### Water repellency

**Figure 3:**
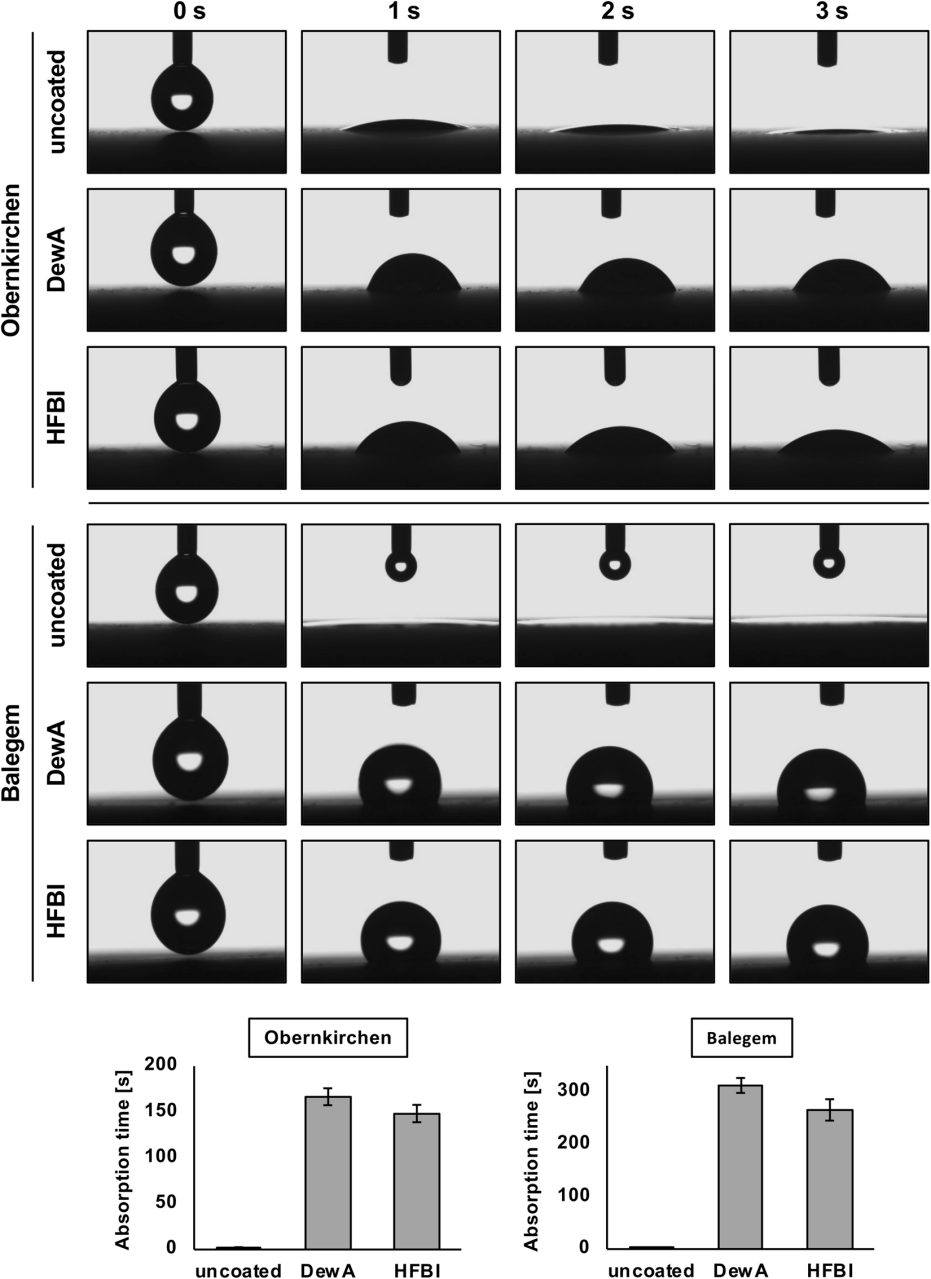
Reduced water absorption of hydrophobin coated stone. Water droplets were applied on the stone surface and absorption of the droplets was documented with a camera until complete absorption. Single frames from the droplets touching the stone surface are shown after 1 s, 2 s and 3 s contact. Data for absorption time are shown as mean ± standard deviation (n = 5).

**Figure 4:**
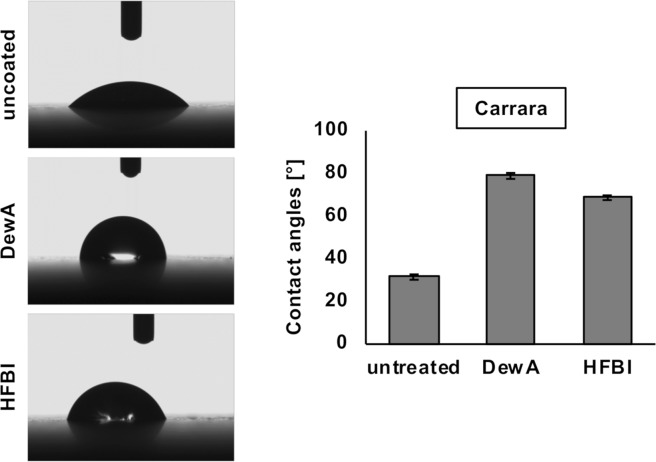
Water contact angles of DewA and HFBI treated Carrara marble. Deionized water droplets were put on the marble surface and imaged with a CCD camera. The results of the water contact angles are shown as mean ± standard deviation (n = 10).

To test if the hydrophobin coatings can confer hydrophobicity to different lithotypes, droplets of deionized water were put on top of the treated samples and water absorption was followed visually. The droplets remained on the surface for 2.3 ± 0.2 s on untreated Obernkirchen and for 1.7 ± 0.5 s on untreated Balegem. On DewA-coated Obernkirchen, the droplets remained on the surface for 166.7 ± 9.2 s, whereas on HFBI coated sandstone the droplets stayed for 148.3 ± 9.4 s. Balegem coated with DewA showed the strongest water repellency with droplet retention times of 312.3 ± 14.3 s, reduced to 265.7 ± 20.4 s for HFBI (Fig. 3). White Carrara marble did not absorb water droplets. Water contact angles were calculated to further characterize hydrophobin coatings. Contact angles of untreated marble were 31.4 ± 1.3°. The measured contact angles for DewA-coated marble were 78.8 ± 1.4° and for HFBI 68.6 ± 1.1° (Fig. 4). Because the droplets on non-treated stones of the other two lithotypes were absorbed so quickly contact angles could not be determined. Generally, both hydrophobins rendered the three tested lithotypes hydrophobic, with DewA being more effective than HFBI.

### Water evaporation

**Figure 5:**
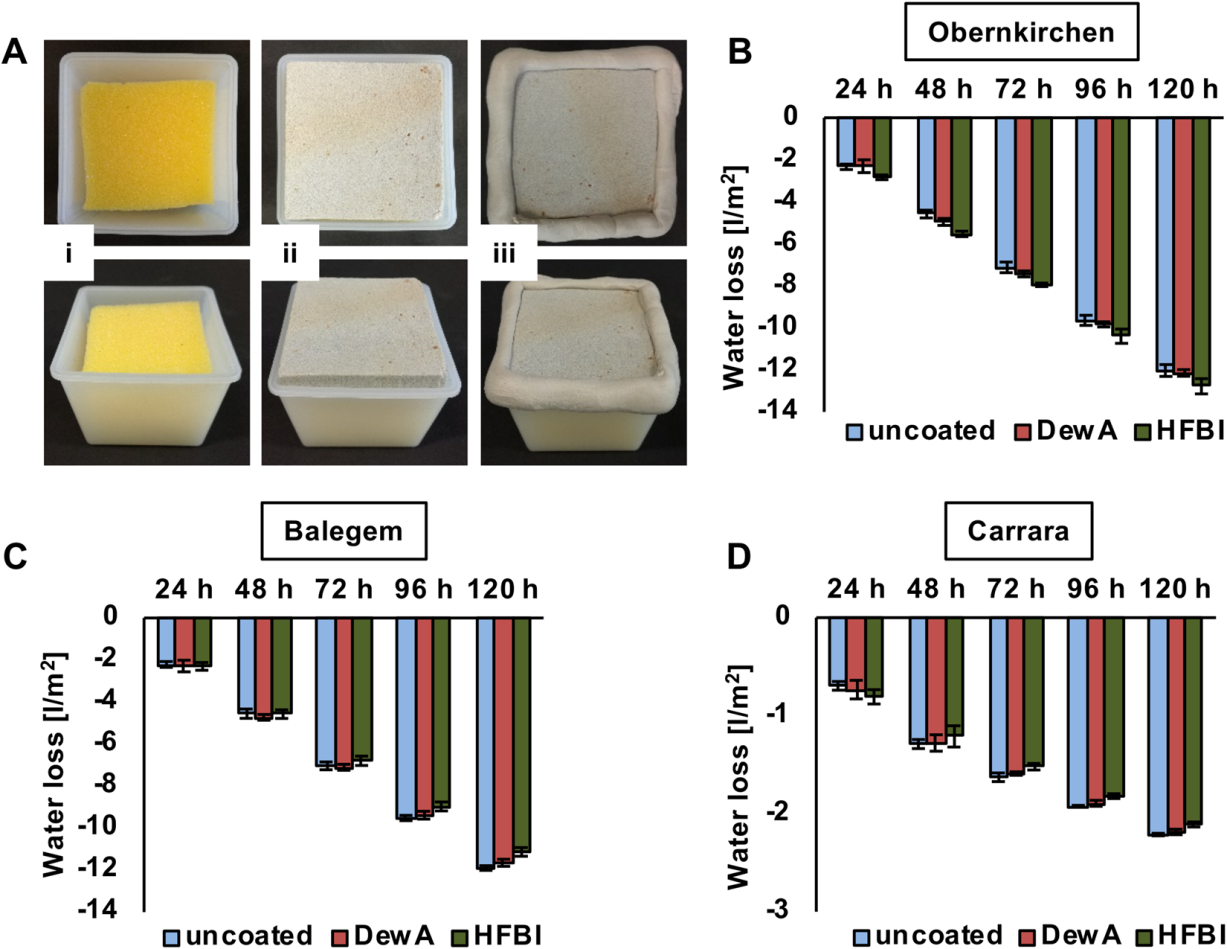
Water evaporation assay. (**A**) Experimental setup. Stone samples were placed on top of a sponge sitting in a small box filled with water (i, ii). Remaining gaps were sealed with sealing compound (iii) and weight loss of the specimens was documented over five days. Boxes are shown from the top (upper row) and from the side (lower row). The amount of evaporated water, which is equal to the weight loss, is shown for Obernkirchen sandstone (**B**), Balegem limestone (**C**) and Carrara marble (**D**). Data shown as mean ± standard deviation (n = 5).

An important aspect of stone protection is the ability of water permeation even through coated stones to allow transpiration and therefore avoidance of water inclusion. With hydrophobins DewA and HFBI treated 5 cm × 5 cm × 1 cm stone samples were placed with the uncoated side on a sponge placed in a container filled with deionized water. The gap between stone and container was sealed air-tight with plastic-fermit and weight loss of the specimens was documented for 5 days (Fig. 5A). The untreated Obernkirchen sandstone lost 12.03 l/m^2^ water by evaporation whereas the samples coated with DewA (12.15 l/m^2^) and HFBI (12.78 l/m^2^) even lost slightly more water than the control (Fig. 5B). The untreated Balegem specimens lost 11.9 l/m^2^, the DewA coated 11.65 l/m^2^ and the HFBI coated 11.13 l/m^2^, which corresponds to 6.5% less evaporation compared to the uncoated samples (Fig. 5C). The marble specimens lost 2.21 l/m^2^ (untreated), 2.18 l/m^2^ (DewA coated) and 2.11 l/m^2^ (HFBI coated) by evaporation (Fig. 5D). The differences between untreated and hydrophobin coated stones were not significant.

### Coating stability

**Figure 6:**
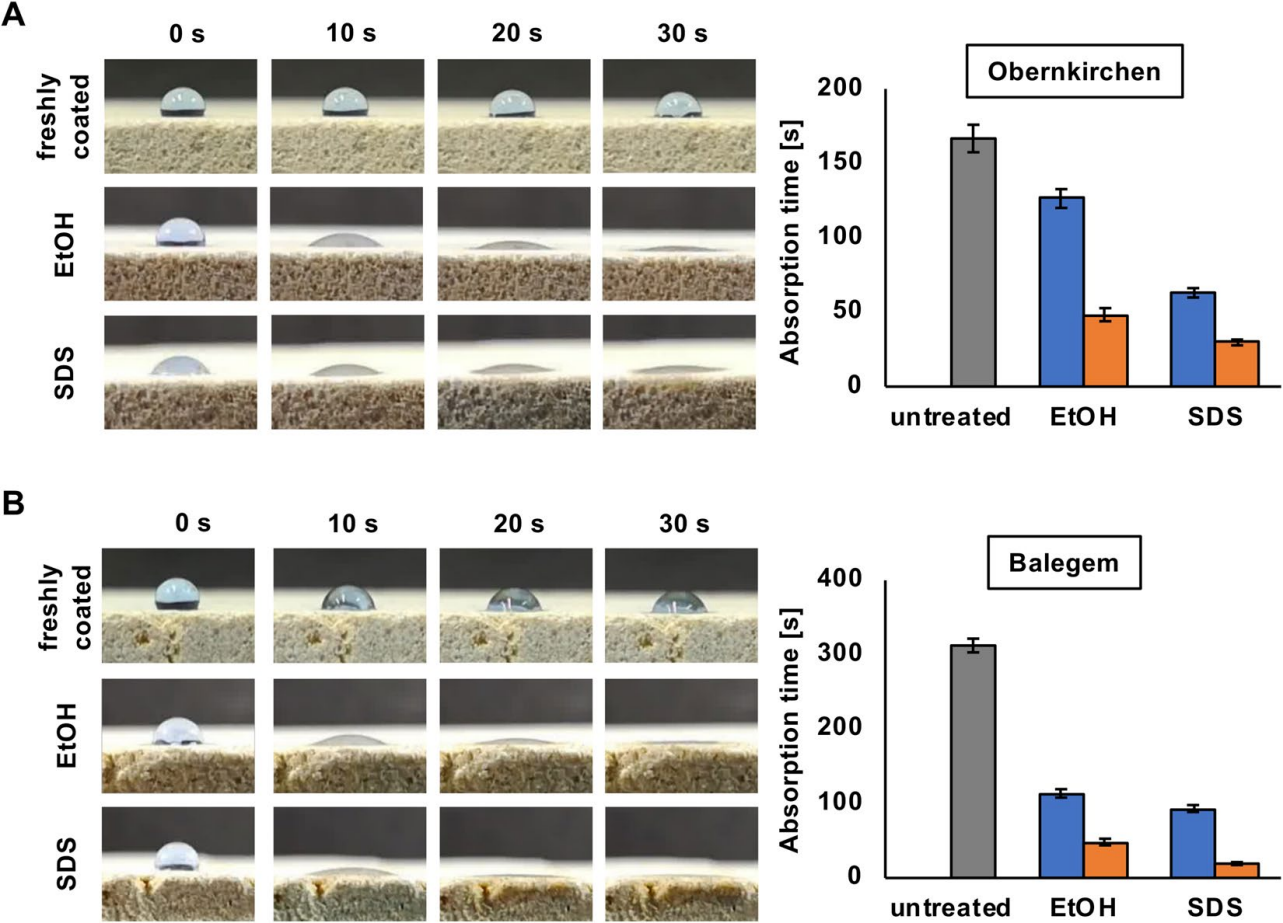
Reversibility of hydrophobin coating. DewA coated Obernkirchen sandstone (**A**) and Balegem limestone (**B**) were incubated for one day (blue) or three days (orange) in 70% ethanol or 1% SDS. Absorption of water droplets was measured after the treatment and compared with the “untreated” data. Data shown as mean ± standard deviation (n = 3).

**Figure 7:**
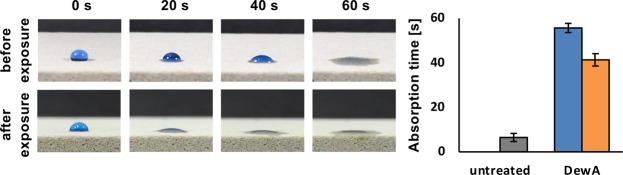
Stability assay. DewA coated Obernkirchen sandstone was exposed for three months outside (at cologne cathedral or a rooftop near Karlsruhe, Germany) and water absorption documented before (blue) and after (orange) exposure. Data shown as mean ± standard deviation (n = 3).

With DewA showing the best results in water repellency, the stability of the DewA hydrophobin coating on Obernkirchen sandstone and Balegem limestone was tested against alcohol (70% EtOH) and detergent (1% SDS). DewA-coated stone specimens were put in aqueous solutions of 70% EtOH or 1% SDS and water repellency was analyzed after 1 and 3 days, respectively. For Obernkirchen, the droplet absorption time was reduced to 126.3 ± 8.7 s after 1 day in 70% EtOH and to 63.0 ± 3.9 s after 1 day in 1% SDS. After an incubation for 3 days, absorption times were reduced to 48.3 ± 4.4 s for EtOH and to 29.7 ± 1.9 s for the SDS incubated samples (Fig. 6A). After 1 day, the droplet absorption time for Balegem was 113.7 ± 7.6 s for the sample in 70% EtOH and 93.3 ± 5.7 s for the sample incubated in 1% SDS. After 3 days, the absorption time was 48.3 ± 4.4 s for the alcohol specimen and 19.3 ± 2.4 s for the SDS one (Fig. 6B). Additionally, cubic specimens with 5 cm side length were treated with DewA and exposed outside on a rooftop to assess the coating stability under natural weather conditions. The freshly coated specimens showed an absorption time of 55.7 ± 21 s. After 3 months, the absorption time was reduced by 25.9% to 41.3 ± 2.8 s (Fig. 7).

## Discussion

DewA and HFBI, typical representatives of class I and class II hydrophobins, were able to coat and to reduce the water absorption of different lithotypes, independent of their chemical nature and structure. Especially DewA generated a strong water repellency on all three lithotypes without decreasing the vapor permeability of the stone samples. The coating with hydrophobin, in comparison to various traditional treatments^5,32^, has not clogged the stone pores and allowed water evaporation through the stone. This feature is crucial for the protection of architectural heritage buildings, since water that accumulates inside the stone bulk will cause severe damage to the material. It has been shown before that hydropobin layers are permeable for vapor^31^. However, in the case of stones it is conceivable that the pores remain open because of their diameter and are not sealed with hydrophobin layers. The nanoparticle-sized hydrophobins were able to penetrate up to 2 cm in porous sand- and limestone specimens. Compared to traditional protective and consolidation formulations that only reach from a few mm up to 1 cm^33–36^, this is quite deep. This may be due to the characteristics of hydrophobins to change the polarity of coated surfaces and to reduce the water surface tension, both crucial parameters causing strong capillarity forces^37^. The hydrophobin coating was easily removable with alcohol and detergent, the main components of cleaning agents. This is in line with the restorer charter pointing out that every newly developed protective must be reversible, meaning removable from the stone surface without damaging the substrate^26,27^. As a protein, the long-term stability of hydrophobins when exposed in the environment seems however limited. Comparing the water contact angles of DewA coated marble (79°) with the results achieved by the commercially available product Silres® BS29A (140°)^38^, the use of hydrophobins as sole water-repellent protective seems not favorable. Another potential usage of hydrophobins in the field of stone consolidation could be their use together with consolidation products or as a pretreatment to enhance the penetration depth of the applied product. Also, the functionalization of DewA with small peptides that improve the polymerization of diammonium phosphate to hydroxyapatite is possible^39^. (NH_4_)_2_HPO_4_ reacts with calcium to solid hydroxyapatite and is widely used as consolidation product^35,40,41^. Even the fusion of antimicrobial peptides to hydrophobins is possible and could reduce microbial growth on stone surface^42^. This study provides first insights in the potential use of hydrophobins in stone conservation and may be a stimulus for further investigations in that aspect.

## Methods

### Lithotypes and hydrophobin production

Obernkirchen sandstone from Lower Saxony, Germany, is mainly composed of quartz and kaolinite and has an open porosity of 24.1%. Balegem limestone is a sedimentary rock with large siliceous clasts in a calcitic matrix with a compact texture and open porosity of 9.9%, found around Ghent, Belgium. Carrara marble is a metamorphic rock with almost purely calcific composition and highly compact texture with an open porosity of 0.7%. Hydrophobins DewA and HFBI were produced as described previously^30^. Shortly, DewA and HFBI, carrying a 6xHis tag, were expressed in *E. coli*, purified from inclusion bodies and freeze dried for long term storage. Freshly prepared solutions of 100 µg/ml hydrophobin in deionized water were used for stone coating.

### Stone coating

Before coating, stone specimens were incubated for 6 hours in deionized water to remove residual salts and dried for 36 hours at 60 °C. Stone plates were placed 5 mm deep in 100 µg/ml DewA or HFBI solutions and incubated at 60 °C for two hours. 100 µg/ml hydrophobin were shown to form uniform monolayers/coatings on water-gas interfaces as well as on hard surfaces^30,31^. After three washing steps with deionized water for 15 minutes, the stones were dried over night at room temperature.

### Immunodetection

For specific immunodetection of hydrophobin, untreated and hydrophobin-coated stone samples were blocked for 30 min with 10% milk in TBS (Tris-buffered saline) at room temperature. The primary α-His antibody (Thermo Fisher Scientific, Waltham, USA), diluted 1:3000 in 1% milk in TBS was applied for 1 hour. The stones were washed three times for 5 minutes in TBS and the secondary HRP-labelled antibody (Sigma-Aldrich, Hamburg, Germany) was applied in a 1:10000 dilution in 1% milk in TBS for one hour. After several washing steps in TBS and water, immunodetection was carried out with WesternBright ECL HRP solution (Advansta, Menlo Park, USA).

### Determination of water absorption

8 µl water droplets colored with Remazol Brilliant Blue were applied on the stone surface and imaged with a camera. The time until the complete drop was absorbed in the stone was measured.

### Determination of water contact angles

The static water contact angles of uncoated and coated marble were measured with an OCA20 and the software SCA 202 v3.12.11 (both DataPhysics Instruments GmbH). 4 μl deionized water droplets were put on the surfaces and imaged with a CCD camera (resolution of 768 × 576 px). An ellipse fit was chosen to approach the droplet form, followed by the determination of the contact angles.

### Water evaporation

To measure the water evaporation of coated stone, a water-soaked sponge was placed in a box and the pot was filled half with deionized water. The stone samples were placed on top of the sponge and the remaining gap between stone and pot was closed with sealing compound. The weight loss of the specimen, equal to the amount of water evaporated through the stone, was monitored for five days on a micro scale. Statistics were carried out using the two-tailed student’s t-test assuming equal variance with an alpha level of 0.05. Data were tested for normality using the Anderson-Darling test.

### Coating stability

Freshly coated stones were incubated for one or three days in alcohol (70% EtOH) and detergent (1% SDS), the main components of cleaning agents. After several rinsing steps with deionized water and complete drying of the specimens, the water absorption was measured to determine the reduction in water repellency, indicating the removal of the hydrophobins from the stone. For long term stability, DewA coated Obernkirchen sandstone cubes with 5 cm side length were exposed outside at the Cathedral Church of Saint Peter (Cologne, Germany) and a rooftop near Karlsruhe, Germany. The water absorption was measured before and after the exposure.
